# The role of social connection on the experience of COVID-19 related post-traumatic growth and stress

**DOI:** 10.1371/journal.pone.0261384

**Published:** 2021-12-15

**Authors:** Marcela Matos, Kirsten McEwan, Martin Kanovský, Júlia Halamová, Stanley R. Steindl, Nuno Ferreira, Mariana Linharelhos, Daniel Rijo, Kenichi Asano, Sara P. Vilas, Margarita G. Márquez, Sónia Gregório, Gonzalo Brito-Pons, Paola Lucena-Santos, Margareth da Silva Oliveira, Erika Leonardo de Souza, Lorena Llobenes, Natali Gumiy, Maria Ileana Costa, Noor Habib, Reham Hakem, Hussain Khrad, Ahmad Alzahrani, Simone Cheli, Nicola Petrocchi, Elli Tholouli, Philia Issari, Gregoris Simos, Vibeke Lunding-Gregersen, Ask Elklit, Russell Kolts, Allison C. Kelly, Catherine Bortolon, Pascal Delamillieure, Marine Paucsik, Julia E. Wahl, Mariusz Zieba, Mateusz Zatorski, Tomasz Komendziński, Shuge Zhang, Jaskaran Basran, Antonios Kagialis, James Kirby, Paul Gilbert

**Affiliations:** 1 University of Coimbra, Center for Research in Neuropsychology and Cognitive Behavioral Intervention (CINEICC), Coimbra, Portugal; 2 Centre for Compassion Research and Training, College of Health, Psychology and Social Care, University of Derby, Derby, United Kingdom; 3 Faculty of Social and Economic Sciences, Institute of Social Anthropology, Comenius University, Bratislava, Slovakia; 4 Faculty of Social and Economic Sciences, Institute of Applied Psychology, Comenius University, Bratislava, Slovakia; 5 Compassionate Mind Research Group, School of Psychology, The University of Queensland, Brisbane, Australia; 6 Department of Social Sciences, University of Nicosia, Nicosia, Cyprus; 7 Department of Psychological Counseling, Faculty of Psychology, Mejiro University, Tokyo, Japan; 8 Department of Psychology, Faculty of Biomedical and Health Sciences, Behavior, Emotions, and Health Research Group, Universidad Europea de Madrid, Madrid, Spain; 9 Escuela de Psicología, Pontificia Universidad Católica de Chile, Santiago, Chile; 10 Evaluation and Treatment in Cognitive and Behavioral Psychotherapies—Research Group (GAAPCC), Pontifical Catholic University of Rio Grande do Sul, Porto Alegre, Brazil; 11 Conectta: Mindfulness & Compassion, São Paulo, Brazil; 12 Motivación Compasiva, Buenos Aires, Argentina; 13 Neuroscience Department, Section of Psychiatry and Psychology, King Faisal Specialist Hospital and Research Centre (KFSH&RC), Jeddah, Saudi Arabia; 14 School of Human Health Sciences, University of Florence, Florence, Italy; 15 Department of Economics and Social Sciences, John Cabot University, Rome, Italy; 16 Center for Qualitative Research in Psychology and Psychosocial Well-being, National and Kapodistrian University of Athens, Athens, Greece; 17 Department of Educational and Social Policy, University of Macedonia, Thessaloniki, Greece; 18 Mindwork Psycological Center, Copenhagen, Denmark; 19 Department of Psychology, University of Southern Denmark, Odense, Denmark; 20 Department of Psychology, Eastern Washington University, Cheney, WA, United States of America; 21 Department of Psychology, University of Waterloo, Waterloo, Canada; 22 Laboratoire Inter-universitaire de Psychologie: Personnalité, Cognition et Changement Social, Grenoble Alpes University, Grenoble, France; 23 Centre Hospitalier Alpes Isère, C3R - Réhabilitation psychosociale et remédiation cognitive, Grenoble, France; 24 CHU de Caen, Service de Psychiatrie Adulte, Caen, France; 25 UNICAEN, ISTS, GIP Cyceron, University of Normandy, Caen, France; 26 The Mind Institute Poland, Warsaw, Poland; 27 SWPS University of Social Sciences and Humanities, Warsaw & Poznań, Poland; 28 Department of Cognitive Science, Nicolaus Copernicus University, Torún, Poland; 29 Neurocognitive Laboratory, Centre for Modern Interdisciplinary Technologies, Nicolaus Copernicus University, Torún, Poland; 30 School of Human Sciences, University of Derby, Derby, United Kingdom; Santa Lucia Foundation, IRCSS, ITALY

## Abstract

**Background:**

Historically social connection has been an important way through which humans have coped with large-scale threatening events. In the context of the COVID-19 pandemic, lockdowns have deprived people of major sources of social support and coping, with others representing threats. Hence, a major stressor during the pandemic has been a sense of social disconnection and loneliness. This study explores how people’s experience of compassion and feeling socially safe and connected, in contrast to feeling socially disconnected, lonely and fearful of compassion, effects the impact of perceived threat of COVID-19 on post-traumatic growth and post-traumatic stress.

**Methods:**

Adult participants from the general population (*N* = 4057) across 21 countries worldwide, completed self-report measures of social connection (compassion for self, from others, for others; social safeness), social disconnection (fears of compassion for self, from others, for others; loneliness), perceived threat of COVID-19, post-traumatic growth and traumatic stress.

**Results:**

Perceived threat of COVID-19 predicted increased post-traumatic growth and traumatic stress. Social connection (compassion and social safeness) predicted higher post-traumatic growth and traumatic stress, whereas social disconnection (fears of compassion and loneliness) predicted increased traumatic symptoms only. Social connection heightened the impact of perceived threat of COVID-19 on post-traumatic growth, while social disconnection weakened this impact. Social disconnection magnified the impact of the perceived threat of COVID-19 on traumatic stress. These effects were consistent across all countries.

**Conclusions:**

Social connection is key to how people adapt and cope with the worldwide COVID-19 crisis and may facilitate post-traumatic growth in the context of the threat experienced during the pandemic. In contrast, social disconnection increases vulnerability to develop post-traumatic stress in this threatening context. Public health and Government organizations could implement interventions to foster compassion and feelings of social safeness and reduce experiences of social disconnection, thus promoting growth, resilience and mental wellbeing during and following the pandemic.

## Introduction

The rapid spread of COVID-19 around the world brought with it unprecedented psychosocial stresses that impact on mental health [1–3]. The psychological impact is unprecedented because the threat from COVID-19 is continuous, invisible and universal [4, 5]. In a review of the impact of COVID-19 on mental health, Vindegaard and Benros [6] found greater anxiety and depression in the general public; increased depression, anxiety, psychological distress and poor sleep quality in healthcare professionals; and high levels of depression and post-traumatic stress symptoms in patients who had experienced COVID-19. Another systematic review reported high prevalence of post-traumatic stress disorder (PTSD) symptoms related to the COVID-19 pandemic among health care workers and identified a lack of social support as potential predictor [7]. Epidemiological studies have also documented that 17% of adults in the general population experienced PTSD symptoms during the early stages of the pandemic [8]. In fact, it has been argued that, due to the nature of the pandemic threat, exposure to the COVID-19 pandemic and its associated health, psychological, social, and economic consequences, can constitute a traumatic event as described in classification systems like the ICD-11 [9, 10].

While a traumatic event can cause post-traumatic symptoms, it can also be a catalyst for positive change, with mounting research showing post-traumatic growth resulting from an adaptive response to, and coping with, trauma [11, 12]. Despite the negative sequelae of COVID-19 on mental health, research begun documenting positive psychological effects of the pandemic. For example, increased post-traumatic growth was reported by carers of children in Portugal and the UK and was associated with higher levels of wellbeing [13]. Similarly, and moderate levels of post-traumatic growth were found in frontline nurses and were related to social support [14]. Perceived social support, along with regulatory emotional self-efficacy, were also found to mediate the link between emotional creativity and posttraumatic growth during the COVID-19 crisis [15].

Whether individuals experience mental health difficulties and post-traumatic stress or experience resilience and growth (post-traumatic growth) in response to traumatic events may depend on individual coping styles. For example, suspiciousness, intolerance of uncertainty, anxiety about death [16] and negative rumination [17] were associated with developing mental health difficulties (PTSD symptoms in particular). In contrast, beliefs about a good world, openness to the future, identification with humanity [16] and constructive reflection (i.e., thinking of solutions) [17] were associated with post-traumatic growth. Indeed, when controlling for a range of variables (e.g., psychological distress, perceived social support, age, gender, ethnicity, and education) the only significant predictor of post-traumatic growth was social support [18].

### Social connection

Having access to caring, supportive social connections has a range of benefits for mental and physical health [19–21] and is negatively linked to depression, anxiety [22, 23] and post-traumatic stress [24]. In regard to major disasters, that affect groups and populations, social support is a strong predictor for how people cope with adversity, and is associated with increased resilience and post-traumatic growth [25, 26]. In a review by Saltzman, Hansel and Bordnick [27], which examined a range of large-scale disasters such as Hurricane Katrina, floods, earthquakes and mining disasters, the role of social support was shown to be crucial to people’s abilities to cope, recover and prevent mental health difficulties. In other words, how people turn to each other and feel supported by each other, is central to people’s ability to adaptively respond to disasters. In addition, feeling socially safe is positively linked to feeling socially connected to others, supported in close social relationships and being resilient, and is negatively linked to depression and anxiety [22, 23]. Social safeness is associated with decreased traumatic impact of early adverse events and to mediate the link between early emotional trauma and depressive symptoms [28]. Feelings of ‘social safeness’ may be an emotion regulation process in its own right that can be distinguished from positive affect and negative affect, and are a unique predictor of stress (Armstrong et al., 2020), which might act as a buffer against mental health problems. Social safeness is linked to being open and receptive to support and compassion from others [29–31].

Therefore, in the face of adversity, having a sense of social connection and affiliation is a critical psychosocial strategy for promoting adjustment, resilience, and growth [21]. In fact, humans evolved as a highly social species, innately motivated to seek and respond to attachment to carers [32, 33] and to form and maintain positive and significant social relationships [34, 35]. The evolution of caring has led to adaptations to the central and autonomic nervous systems (e.g., the myelination of the vagus nerve), that facilitated the co-evolution of care-providing and care-seeking forms of relating, and played a significant role in the regulation of threat and the soothing qualities of social connection [29, 36–38]. Caring social connections are hence powerful psychological and physiological regulators. They significantly impact on a range of physiological systems (e.g., brain development, autonomic, neuroendocrine and immune functions, gene expression) [21, 35–38], and have emotional and self-regulating properties [39]. The presence of social safeness and connection not only downregulates physiological arousal, but also stimulates the oxytocin-opiate circuits and the vagus nerve, and has soothing and/or encouraging qualities [35, 37]. In addition to providing important coping resources (e.g., social support), which can help protect against the harmful effects of adversity and even promote psychosocial growth [29, 40, 41], social connection can shift the body biological patterns away from a threat-related physiological arousal and pro-inflammatory state (e.g., associated with reduced risk for anxiety, depression, cardiovascular disease, autoimmune disorders), toward a higher vagal tone physiological state (e.g., related to greater capacity for emotion regulation, metacognitive awareness, empathy, prosociality, or to the inhibitory function of the prefrontal cortex; [36, 39, 42] and more anti-viral state (e.g., linked to decreased susceptibility to viruses; [21, 41, 43].

There are, however, different dimensions to experiencing caring social connections, one of which is compassion [29, 31]. Compassion can be defined in various ways [44, 45], but in evolutionary focused models it has been conceptualized as a prosocial motivation and defined as the sensitivity to suffering in self and others with a commitment to try to alleviate or prevent it [46]. In light of this definition there are two major components of compassion: the preparedness to engage with suffering and distress, and the wisdom to work out helpful action.

Compassion is proposed to have evolved from the mammalian care-giving systems, and comprises a range of physiological and emotional regulating systems, specifically designed for down-regulating threat and allowing states of ‘*rest and digest’* [36, 37]. Evolved physiological (e.g., the myelinated vagus nerve, oxytocin) and psychological mechanisms (e.g., social competencies and intelligence) that underpin caring motives and behaviour [37, 47] are at the root of compassion. In particular, compassion emerges from the blend of an innate mammalian caring motivation (to detect and respond to the needs of dependent offspring) and complex human cognitive competencies (e.g., mind awareness, empathic awareness, social intelligence, knowing intentionality), which have evolved over the last two million years and transform basic caring motives into potentials for compassion [35, 48, 49]. Compassion can therefore be helpful in the face of adverse events such as the COVID-19 pandemic, as it involves a sensitivity to the suffering (in oneself and others), the preparedness and courage to engage with that suffering and tolerate distress, the wisdom to reflect on the nature and causes of suffering and to recognize it as part of a larger shared human experience, along with a deep commitment to work out helpful action in an effort to prevent or alleviate that suffering [49]. By doing so, compassion can help to downregulate threat emotional states while facilitating access to the safeness-soothing affect regulation system, and thus fostering one’s sense of safeness, connectedness, positive affect and self-soothing abilities [49].

Compassion operates as a dynamic intra- and interpersonal process that unfolds in a social interactional context and can be seen as a flow, whereby we can be compassionate to others, be compassionate to ourselves and also be open to receiving compassion from others [49, 50]. These multidimensional flows of compassion are also protective factors against psychological distress [50–53]. Treating oneself and others compassionately is associated with resilience, mental and neurophysiological wellbeing and prosocial behaviour [39, 44, 50, 54–59]. Being open and responsive to receiving compassion from others is negatively associated with symptoms of depression, anxiety and stress and positively associated with wellbeing [50], and buffers the effect of self-criticism on depression [60]. Additionally, self-compassion has been established by extensive literature as a buffer against psychological distress (see [61] for a review). Hence receiving compassion (from others and from oneself) can act as a protective factor during difficult times. In the context of traumatic events, self-compassion has been linked to greater post-traumatic growth [62, 63], and associated with less post-traumatic stress symptomatology, with tentative evidence suggesting that compassion interventions potentially reduce PTSD symptoms [64]. It has been suggested that some of the possible mechanisms between the protective effects of social support and compassion on reduced PTSD might be lower psychological inflexibility [65], emotional dysregulation [66] and avoidance strategies [67]. In the context of COVID-19, both self-compassion as a unidimensional construct [68–70], and the flows of compassion as a multidimensional construct [71] have been found to be protective factors against psychological distress. In particular, compassion for self buffered the effects of the perceived threat of COVID-19 on psychological distress, whereas compassion from others alleviated the impact of fears of contracting COVID-19 on social safeness [72].

### Social disconnection

In contrast to feeling socially safe and connected to others and being able receive and give compassion, people can feel socially disconnected and lonely, and be fearful of compassion. With the evolution of the mammalian care-giving system, humans rely on social relations as physiological and emotional regulators, indeed painful feelings associated with social disconnection, rely on the same neurological processes that underlie experiences of physical pain [73]. Social disconnection has been identified as increasing the risk of mental and physical health difficulties [74]. A meta-analysis found that as a negative emotion which tends to be chronic and recurrent, loneliness was a significant variable affecting depression [75]. Possible mechanisms between social disconnection and health risks include, their shared neurological basis with physical pain [73] and appraising everyday events as more intensely stressful and passive coping with said stressors [76].

During the COVID-19 pandemic, beyond the threat of being contaminated with the virus, spreading the virus to family, friends, and vulnerable people, lockdown actions taken by governments in an effort to contain the virus also had a significant impact on mental health through physical entrapment inside homes [1] and reducing opportunities for social support [77]. Lockdown was found to increase experiences of depression, anxiety, stress and social disconnection and loneliness [1, 2, 27, 78–81]. While physical loneliness is an obvious issue in the pandemic [2], emotional loneliness where individuals feel emotionally disconnected and unable to share difficult emotions and experiences or gather support, plays a central role in coping with adversity. As Saltzman et al. [27] note:

During this pandemic, the messaging has also had a negative impact in reinforcing the “you’re alone or isolated” theme. For example, the term “social distancing” has been a constant call-to-action on TV, radio, and social media versus the more appropriate term “physical distancing,” adding to the perception of isolating oneself socially (p.55).

Unique to this pandemic has been it depriving people of the very thing they need (i.e., social support) in order to become resilient and adaptively cope with adversity. This is in stark contrast to previous disasters where social support was found crucial to protect mental health and promote resilience and post-traumatic growth [26, 27]. Social isolation has indeed been found to stimulate midbrain craving responses, similar to hunger, associated with the craving of social interactions [82].

Moreover, some individuals can develop and experience fears of receiving and giving compassion [83, 84], being unable to activate compassionate motivational systems or use caring relationships as affect regulators [85]. Fears of compassion can be experienced across the three flows (i.e., for others, from others, for self), and are understood as inhibitors that hinder compassionate motivation of being ‘turned-on’ or ‘acted on’, because the signal of suffering is either not noticed/avoided or does not result in an action to prevent or alleviate that suffering. Fears of compassion may, for example, be linked to the belief that compassion is a self-indulgence or a weakness, that if compassionate (to oneself or others) one will become too distressed or unable to cope, or that oneself or others are not deserving of compassion [84]. Thus, fears of compassion inhibit one’s ability to activate compassion across the three flows which negatively affects physiological and psychological health and wellbeing [86]. There is now considerable evidence documenting that fears of compassion, especially of self-compassion and of receiving compassion from others, are strongly linked to problems of depression, anxiety and stress, and to vulnerability factors, such as self-criticism and shame [84, 87]. Fears of compassion for the self and from others were associated with the traumatic impact of early emotional experiences and were significant mediators of the impact of adverse events on depression and anxiety symptoms, and on paranoid ideation about other people as potential threats [88].

In the context of traumatic events, lower fears of self-compassion were associated with less PTSD symptomatology [64]. In a multinational study during the COVID-19 pandemic, Matos et al. [72] found that all the flows of fears of compassion magnified the impact of perceived threat of COVID-19 on psychological distress, but only fears of compassion from others amplified the effect of the perceived likelihood of contracting the virus on how socially safe people felt.

### Aims

Given the need to examine both protective and risk factors associated with the negative and positive psychological consequences of the current global COVID-19 pandemic [1, 89], the current study examines how dimensions of social connection (i.e., the compassion flows and social safeness) and social disconnection (i.e., fears of compassion and loneliness) relate to post-traumatic growth and post-traumatic stress during the early months of the COVID-19 pandemic in a global adult population across 21 countries. We hypothesised that post-traumatic stress and growth would be impacted by the degree to which individuals feel socially safe, connected and open to compassion, or disconnected, lonely and fearful of compassion.

Specifically, this study aims to examine whether the dimensions of social connection (i.e., the compassion flows and social safeness) and social disconnection (i.e., fears of compassion and loneliness) moderate the impact of perceived threat of COVID-19 (i.e., fear of contracting SARS-Cov-2) on post-traumatic growth and on post-traumatic stress symptoms. It was hypothesised that the social connection component (i.e., the compassion flows and social safeness) would magnify the effects of perceived threat of COVID-19 on post-traumatic growth (i.e., recovery and growth), and, conversely, that the social disconnection component (i.e., fears of compassion and loneliness) would magnify the impact of perceived threat of COVID-19 on post-traumatic stress symptoms.

## Materials and methods

### Participants

The research sample comprised of 4057 participants from 21 countries: Argentine (ARG) *N* = 257, Australia (AUS) *N* = 109, Brazil (BRA) *N* = 299, Canada (CAN) *N* = 115, Chile (CHL) *N* = 282, China (CHN) *N* = 77, Columbia (COL) *N* = 50, Cyprus (CYP) *N* = 38, Denmark (DNK) *N* = 141, France (FRA) *N* = 115, Great Britain (GBR) *N* = 268, Greece (GRE) *N* = 145, Italy (ITA) *N* = 160, Japan (JPN) *N* = 522, Mexico (MEX) *N* = 181, Poland (POL) *N* = 82, Portugal (PRT) *N* = 394, Saudi Arabia (SAU) *N* = 256, Slovakia (SVK) *N* = 46, Spain (ESP) *N* = 392, and The United States of America (USA) *N* = 128. The sample consisted of 18.2% of males and 80.8% of females, 0.4% of participants reported other gender and 0.6% preferred not to report their gender. The mean age of the sample was 41.45 years old (*SD* = 14.96). In regard to respondents’ experience of COVID-19 disease, 94.1% (*n* = 3817) reported not having been infected with COVID-19, 2% (*n* = 80) indicated having contracted the disease, 2.9% (*n* = 117 from Brazil) responded they didn’t know, 0.9 (*n* = 37) preferred not to respond and 6 participants did not respond.

### Measures

The online survey collected sociodemographic information (nationality, country of residence, age, gender), COVID-19 related information (e.g., experience of COVID-19 infection) and administered self-report instruments assessing dimensions of social connection (i.e., compassion for self, from others, for others, and social safeness), dimensions of social disconnection (i.e., fears of compassion for self, from others, for others, and loneliness), perceived threat of COVID-19, post-traumatic growth and post-traumatic stress symptoms.

#### Social connection

Social connection was measured using the *Compassionate Engagement and Action Scales* and the *Social Safeness and Pleasure Scale*, described below.

*Compassionate Engagement and Action Scales* (CEAS) includes three scales that assess the three flows of compassion: self-compassion, compassion to others and compassion received from others, with 13 items each [50, 51]. For example, “I am motivated to engage and work with my distress when it arises”. Each scale measures different elements of compassion *Engagement* (6 items and 2 filler items) and *Action* (4 items and 1 filler item). Participants are asked to rate each item on a ten-point Likert scale, based on how frequently it occurs, from 1 (never) to 10 (always). Each scale can be analysed in terms of the Engagement and Action components separately or as a single factor. Here we use each of the three flows of compassion as single factor scales. In the original study, the CEAS showed good internal consistencies (Cronbach’s alphas) and temporal reliability [50, 51]. In the present study, internal consistency ranged between good and excellent: Compassion for self-Engagement Cronbach’s α = .74/Action α = .89; Compassion for others-Engagement α = .81/Action α = .88; Compassion from others-Engagement α = .91/Action α = .93.

*Social Safeness and Pleasure Scale* (SSPS) is an 11-item self-report measure that assesses the extent to which people usually experience their social world as safe, warm and soothing and how connected they feel to others [90]. For example, “I feel easily soothed by those around me”. Participants are asked to rate on a five-point Likert scale how often they feel as described in each sentence from 1 (almost never) to 5 (almost all the time). Higher scores represent higher perceived social safeness and connectedness to others. In the original study, internal consistency was excellent (Cronbach’s α = .92). In the present study, internal consistency is excellent (Cronbach’s α = .94).

#### Social disconnection

Social disconnection was measured using the *Fears of Compassion Scales* and the *UCLA Loneliness Scale*, outlined below.

*Fears of Compassion Scales* (FCS) are three scales that assess fears of compassion, one for each flow: 1) fears of feeling and expressing compassion for others (10-items), 2) fears of receiving compassion from others (13-items), 3) fears of compassion for self (15-items). For example, “People will take advantage of me if they see me as too compassionate”. Respondents are asked to rate on a five-point Likert scale how much they agree with each statement, from 0 (don’t agree at all) to 4 (completely agree) [84]. Higher scores represent higher fears of compassion. In the original study, Cronbach’s alphas were .72 for FCS for others, .80 for FCS from others, and .83 for FCS self-compassion [84]. In the current study, internal consistencies ranged between .89 and .95 (FCS self-compassion Cronbach’s α =. 93, FCS compassion for others Cronbach’s α =. 89, FCS compassion from others Cronbach’s α =. 95).

*UCLA Loneliness Scale* (UCLA LS) is a 20-item self-report measure that assesses one’s subjective feelings of loneliness/social isolation [91]. For example, “I have nobody to talk to”. Participants are asked to rate on a 4-point Likert scale how often each sentence is descriptive of them, from 0 (I never feel this way) to 3 (I often feel this way). After conversion of the reverse coded items, higher scores represent more frequent feelings of loneliness/social isolation. In the original study, Russell [91] found the scale’s internal consistency (Cronbach’s alphas) to range between .89 and .94 across all samples. In the present study, Cronbach’s α = .91 for the overall scale.

#### Perceived threat of COVID-19

Perceived threat of COVID-19 was assessed using *The Perceived Coronavirus Risk Scale* (PCRS), which is an 8-item self-report questionnaire that assesses participants’ fear of getting infected with SARS-Cov-2 in two dimensions: Fear of Contraction (affective aspect) and Likelihood of Contraction (cognitive aspect) [92, 93]. For example, “I worry about getting infected with Coronavirus”. Participants are asked to rate on a five-point Likert scale how much they agree with each sentence from 1 (strongly disagree) to 5 (strongly agree). It has one reversed item. Higher scores represent higher perceived threat of COVID-19. In the original study, Kanovsky and Halamová [92] reported internal consistency to be acceptable (Fear of Contraction Cronbach’s α = .72; Likelihood of Contraction α = .71). In this study, internal consistency was acceptable (Fear of Contraction Cronbach’s α = .70; Likelihood of Contraction α = .70).

#### Post-traumatic growth

Post-traumatic growth was measured using the *Post-traumatic Growth Inventory* (PGI). This 21-item self-report measure assesses positive outcomes reported by people who have experienced traumatic events [94]. For example, “I changed my priorities about what is important in life”. This instrument is organized into five subscales that represent Relating to Others, New Possibilities, Personal Strength, Spiritual Change and Appreciation of Life. Participants are asked to rate on a 6-point Likert scale how much they experienced the changes described by each item, from 0 (I did not experience this change as a result of my crisis) to 5 (I experienced this change to a very great degree as a result of my crisis). In this study, participants were asked to rate the degree to which the described change occurred in their life as a result of the COVID-19 pandemic. Higher scores reflect higher benefits as outcomes of coping with a traumatic event. In the original study, Tedeschi & Calhoun [94] found the subscales’ internal consistency to range between good and questionable (New Possibilities Cronbach’s α = .84; Relating to Others α = .85; Personal Strength α = .72; Spiritual Change α = .85; Appreciation of Life α = .67). The internal consistency for the overall scale is good (Cronbach’s α = .90). In the present study, the total score of the PGI was used and showed an adequate internal consistency (Cronbach’s α = .76).

#### Post-traumatic stress

Post-traumatic stress was assessed using the *Impact of Event Scale—Revised* (IES-R). The IES-R is self-report measure that assesses current psychological distressing symptoms due to a specific stressful event, specifically post-traumatic traumatic stress symptoms [95]. For example, “Any reminder brought back feelings about it”. In the current study, participants were asked to answer the IES-R in relation to the COVID-19 pandemic. This 22-item instrument is organized into three subscales that measure post-traumatic stress related to intrusive thoughts, hyperarousal and avoidance symptoms. Participants are asked to rate on a 5-point Likert scale how distressing each difficulty described by the items has been for them, from 0 (Not at all) to 4 (Extremely). Higher scores mean higher distress associated with each item during the past week. In the original study, Weiss & Marmar [95] found the subscales’ internal consistency (Cronbach’s alphas) to range between .87 and .94 for the Intrusion subscale, .84 and .87 for the Avoidance subscale, and .79 and .91 for the Hyperarousal subscale. In the current study, the total of the IES-R revealed an excellent internal consistency (Cronbach’s α = .94).

### Procedures

The current study is part of a broader longitudinal multinational study on compassion, social connectedness and trauma resilience during the COVID-19 pandemic [e.g., 59]. The study was approved by the Ethics Committee of the Faculty of Psychology and Educational Sciences of the University of Coimbra (UC; CEDI22.04.2020) and was conducted in compliance with the 1964 Helsinki Declaration and its later amendments. Local national ethical approval was also obtained whenever necessary. The current analysis used cross-sectional data collected between mid-April 2020 and mid-May 2020, across 21 countries from Europe, (United Kingdom, Portugal, Spain, Italy, France, Greece, Cyprus, Poland, Slovakia, Denmark), North America (USA, Canada), South America (Brazil, Argentina, Chile, Colombia, Mexico), Asia (China, Japan), Oceania (Australia), and Middle East (Saudi Arabia).

An online survey was created by the research team in English and translated to 11 other languages using forward/backward procedures. When there was already a validation of a self-report questionnaire for a particular language/country that version was selected. The surveys were hosted at the University of Coimbra institutional account in the online platform https://www.limesurvey.org/pt/, and a website was created to support the dissemination of the study across countries (https://www.fpce.uc.pt/covid19study/). The study was disseminated through social and traditional media platforms and institutional/professional emailing lists in each country, using snowball sampling. In addition, Facebook ads were used to promote participation among the general population in some countries. Prior to completing the survey, participants were informed about the study aims and procedures, and the voluntary and anonymous nature of participation. Confidentiality of the collected data was assured, and written informed consent was obtained before the completion of the study protocol. The survey was self-paced and about 25min long. There was no payment to participants for completing the survey.

### Data analysis

For statistical analyses, we used the R program version 4.0.3 [96], package “gamlss” [97] for regression analysis. For the multilevel simultaneous principal component analysis, we used the dedicated software described by Ceulemans et al. [98].

Data analyses proceeded in two steps: (1) to reduce the large number of moderator variables, a Multilevel Simultaneous Component Analysis to obtain component scores was conducted; (2) a set of multilevel regression models to test moderator effects were tested. Firstly, to examine the moderator effects of dimensions of social connection and social disconnection, the large number of variables were reduced. Two main moderator effects were hypothesized linked to dimensions of social connection and social disconnection. The social connection component (41 variables in total), measured by the CEAS for self scale (10 variables), CEAS to others scale (10 variables), CEAS from others scale (10 variables), SPSS social safeness scale (11 variables); and the social disconnection component (58 variables in total), measured by the FCS for self scale (15 variables), FCS for others scale (13 variables), FCS from others scale (10 variables), and the UCLA loneliness scale (20 variables).

The structure of the data (individual responses nested in countries) resulted in multivariate two-level data. As mentioned above, the large number of variables that could moderate the effects of the main predictor (perceived threat of COVID-19) had to be reduced to enter a parsimonious regression model. Principal component analysis (PCA) is routinely applied for such cases. However, standard PCA analysis does not take into account the multilevel structure of data and therefore its component scores could be heavily distorted. Timmerman [99] proposed a class of multilevel simultaneous component models (MLSCA). MLSCA has already been used to study cross-cultural differences [100], and has been recently proposed as a concise alternative [98]. We were not particularly interested in the between-model variance (components at the level of countries), but rather in the within-model variance (components at the level of individual respondents). Our aim was to obtain component scores which were unbiased by the multilevel structure of our data and captured as much of the variance in the data as possible. Unlike the between-submodel, the within-submodel accounts for the covariance structure of the variables within the countries.

There were four main steps of an MLSCA analysis [98]: (1) to fit the different MLSCA variants; (2) to select an appropriate model, i.e., to specify optimal number of within-components and the most adequate model variant for the within-part; (3) to discuss the component matrices of the retained solution; (4) to extract the component score(s) for the subsequent regression analysis.

To select the optimal number of components, Ceulemans et al. [98] recommend using the CHull (convex-hull) test [101, 102], which is similar to the widely used scree-test [103], and works well for MLSCA as well [104]. To conduct this test, the percentage of the variance accounted for (VAF), is plotted against a complexity measure (the number of free parameters corrected for the number of observations). Next, the convex hull of this plot is obtained and the solutions that are located on the higher boundary of this convex hull–denoted as the hull solutions–are retained, as they have the best fit versus complexity balance [98].

As far as the social connection component is concerned, the CHull test (see S1 Table) recommended a single principal component, and so did it for the social disconnection component (see S2 Table). Therefore, we could safely extract two component scores representing individual responses–these two component scores take into account multilevel structure of our data and are therefore unbiased with regards to the differences between countries.

We fitted two sets of multilevel regression models: a) with the sum score of the PGI scale (as a measure of post-traumatic growth) as dependent variable; b) with the sum score of the IES-R scale (as a measure of post-traumatic stress) as dependent variable. For each set of models, we have tested the PCRS fear of contraction scale (as a measure of perceived threat of COVID-19) as the predictor / main effect, the social connection and social disconnection component scores extracted from the MSPCA (see above) as predictors/main effects, and their interaction (social connection and social disconnection component scores as moderators). *R*^2^ (‘variance explained’) statistics were used to measure the effect size of the model. To select the appropriate regression models, we performed: a) analysis of (quantile) residuals to assess the goodness of fit of each model [105]; b) likelihood-ratio tests and information criteria AIC and BIC to compare nested models. The multilevel model decomposes variance between fixed effects (moderation effects) and random effects (differences between countries), and thus it controls for differences between countries.

## Results

### Post-traumatic growth

The first model (m1) was the standard multilevel linear model. After checking its residuals, we concluded that they were platykurtic with heavy tails (see S1 Fig). The second model (m2) was identical, but we tried to predict the variance as well (heteroscedastic model). However, problems with kurtosis and heavy tails were not resolved (see S2 Fig). To solve this problem, we have to relax the assumption of exponential family, and look at models which can explicitly model skewness and kurtosis (normal Gaussian models being their special cases), namely generalized additive models [106]. These have extra parameters in addition to standard mean and variance estimation of normal distribution, and these extra parameters account for skewness and kurtosis–Skew Power Exponential distribution. This distribution was introduced by Azzalini [107] as his type II distribution and was further developed by DiCiccio and Monti [108]. The parameter Nu determines the skewness of the distribution with Nu > 0 indicating positive skewness and Nu < 0 negative. The parameter Tau determines the kurtosis of the distribution, with Tau > 2 for platykurtic data and Tau < 2 for leptokurtic. With Nu = 0 and Tau = 2, this distribution is reduced to the standard normal (Gaussian) distribution.

After fitting this model, it was clear that its fit with our data was acceptable (see the residuals of this model S3 Fig). Note that residuals (not dependent variable) should follow normal distribution if the model has an adequate fit: in other words, residuals should have normal distribution even if the dependent variable is skewed and/or kurtotic–this outcome is justification for the explicit modelling of skewness and kurtosis of the dependent variable. If we compare fit of three fitted models, the Skew Exponential Power model outperformed both Gaussian models (see Table 1). The coefficients of best fitting model are presented in Table 2.

**Table 1:** Likelihood-ratio tests and information criteria for the Post Traumatic Growth (PTG) models.

Model	Deviance	χ^2^ (df)	p-value	AIC	BIC	dist	variance
m1	37219	-	-	37271	37434	normal	homoscedastic
m2	37032	187 (5)	< .001	37127	37425	normal	heteroscedastic
**m3**	**36752**	**310 (2)**	**< .001**	**36850**	**37158**	**SEP**	**Heteroscedastic**

*Note*. χ^2^ = chi-square. df = degrees of freedom. AIC = Akaike information Criterion. BIC = Bayes-Schwarz Information Criterion. dist = distribution., SEP = Skew Power Exponential. Best model is displayed in bold.

**Table 2:** The coefficients of best fitting Post Traumatic Growth (PTG) model—the Skew Exponential Power model.

Predictor	β (SE)	p-value	Σ (SE)	ν (SE)	τ (SE)
Intercept	**35.05 (1.60)**	< .001	**3.64 (0.02)**	**0.30 (0.04)**	**1.67 (0.05)**
PTCSFear	**1.77 (0.29)**	< .001	**-0.04 (0.01)**	-	-
SocialConnection	**4.52 (0.34)**	< .001	**0.07 (0.01)**	-	-
SocialDisconnection	-0.26 (0.78)	.737	**-0.04 (0.02)**	-	-
PTCSFear:SocialConnection	**0.60 (0.26)**	.023	**0.02 (0.01)**	-	-
PTCSFear:SocialDisconnection	-0.11 (0.09)	.194	**-0.01 (0.01)**	-	-

*Note*. β = beta coefficient. SE = standard error. Σ (sigma) = variance. ν (nu) = skewness parameter. τ (tau) = kurtosis parameter. Significant effects are displayed in bold.

The main effect of perceived threat of COVID-19 on post-traumatic growth was significant (and positive): fear of contraction increases post-traumatic growth. The main effect of the social connection component on the post-traumatic growth was significant (and positive), which means that compassion across the three flows and social safeness increase post-traumatic growth. The main effect of the social disconnection component on post-traumatic growth was not significant. The interaction effect of perceived threat of COVID-19 and the social connection component was significant and positive, indicating that the three flows of compassion and social safeness significantly moderate (magnify) the impact of fear of contraction on post-traumatic growth, across all countries. The interaction effect of perceived threat of COVID-19 and the social disconnection component was also significant and negative, revealing that fears of compassion and loneliness significantly moderate (reduce) the impact of fear of contraction on post-traumatic growth, across all countries. Of note, there was significant skewness and kurtosis in the dependent variable (parameters Nu and Tau were both significant). Marginal *R*^2^ amounts to 0.23 which means that all predictors account for 23% of variance of post-traumatic growth.

### Post-traumatic stress

The same procedure was followed for post-traumatic stress. The first model (n1), the standard multilevel linear model, displayed a bad fit with data (see S4 Fig): kurtosis and skewness were problematic. The second, heteroscedastic model (n2) did not improve the fit (see S5 Fig). The multilevel heteroscedastic model with Skew Power Exponential distribution (explicitly modelling the skewness and kurtosis) had acceptable fit (see the residuals of this model in S6 Fig). If we compare the fit of the three fitted models, we can see that the Skew Exponential Power model outperformed both Gaussian models (see Table 3). The coefficients of best fitting model are presented in Table 4.

**Table 3:** Likelihood-ratio tests and information criteria for the Post-Traumatic Stress (IESR) models.

Model	Deviance	χ^2^ (df)	p-value	AIC	BIC	dist	variance
n1	35118	-	-	35271	35434	normal	homoscedastic
n2	35032	89 (5)	< .001	35132	35412	normal	heteroscedastic
**n3**	**34752**	**221 (2)**	**< .001**	**34852**	**35158**	**SEP**	**Heteroscedastic**

*Note*. χ^2^ = chi-square. df = degrees of freedom. AIC = Akaike information Criterion. BIC = Bayes-Schwarz Information Criterion. dist = distribution., SEP = Skew Power Exponential. Best model is displayed in bold.

**Table 4:** The coefficients of best fitting Post-Traumatic Stress (IESR) model—the Skew Exponential Power model.

Predictor	β (SE)	p-value	Σ (SE)	ν (SE)	τ (SE)
Intercept	**18.90 (0.51)**	< .001	**2.50 (0.03)**	**0.65 (0.03)**	**0.78 (0.04)**
PTCSFear	**4.59 (0.21)**	< .001	**0.06 (0.01)**	-	-
SocialConnection	**1.01 (0.25)**	< .001	0.01 (0.01)	-	-
SocialDisconnection	**2.22 (0.73)**	.003	**0.31 (0.04)**	-	-
PTCSFear:SocialConnection	0.36 (0.22)	.099	-0.01 (0.01)	-	-
PTCSFear:SocialDisconnection	**0.46 (0.08)**	< .001	**-0.02 (0.01)**	-	-

*Note*. β = beta coefficient. SE = standard error. Σ (sigma) = variance. ν (nu) = skewness parameter. τ (tau) = kurtosis parameter. Significant effects are displayed in bold.

The main effect of perceived threat of COVID-19 on post-traumatic stress was significant (and positive): fear of contraction increases traumatic symptoms. The main effect of the social connection component on post-traumatic stress was significant and positive which means that compassion and social safeness increase traumatic symptoms. The main effect of the social disconnection component on post-traumatic stress was also significant and positive, indicating that fears of compassion (for self, from others and for others) and loneliness increase traumatic stress symptoms. The interaction effect of perceived threat of COVID-19 and the social connection component was not significant. However, the interaction effect of perceived threat of COVID-19 and the social disconnection component was significant and positive revealing that that fears of compassion and loneliness significantly moderate (heighten) the impact of the fear of contraction on post-traumatic stress, across all countries. Note again that there was significant skewness and kurtosis in the dependent variable (parameters Nu and Tau were both significant). Marginal *R*^2^ amounts to 0.39 which means that all predictors account for 39% of variance.

The above reported moderator effects were consistent across all 21 countries as they were not dominated by individual differences between countries in the levels of the perceived threat of COVID-19, dimensions of social connection (compassion flows, social safeness), dimensions of social disconnection (fears of compassion, loneliness), posttraumatic growth and post-traumatic stress.

## Discussion

The COVID-19 pandemic has had severe multifaceted consequences for people’s psychosocial wellbeing and mental health [2, 3, 67], and hence a better understanding of the underlying protective and risk factors of both the negative and positive psychological effects of the pandemic is warranted [1, 74]. In contrast to previous large-scale disasters, the pandemic has been unique in the respect that, due to the restrictions to human interaction imposed by governments, social connection has not been available as a way to cope with this invisible, persistent and global threat. This study therefore compared the moderating effects of dimensions of social connection (i.e., the compassion flows and social safeness) and social disconnection (i.e., fears of compassion and loneliness) on the impact of perceived threat of COVID-19 on either developing post-traumatic growth or post-traumatic stress symptoms in the context of the pandemic.

### Post-traumatic growth

Higher perceived threat of COVID-19 predicted greater post-traumatic growth. This finding was in line with our expectations given that post-traumatic growth has been proposed as a possible positive psychological consequence of encountering a traumatic event [11, 12, 109]. In fact, when it comes to negative events, perceiving an event as traumatic seems to be a prerequisite for growth [110, 111]. Post-traumatic growth has been associated with post-traumatic stress symptoms and can be regarded as a coping effort in the face of enduring distress [112, 113]. It is logical that one would not have post-traumatic growth without the experience of traumatic stress and, whilst several studies have found this association to be positive, others have found it to be negative [114] and suggested this might be due to how this construct was measured and the dimensions underpinning post-traumatic growth [113]. In support of our findings, recent research has also reported the presence of post-traumatic growth in the context of current threat of COVID-19 [e.g., 13, 14, 16].

Social connection was a significant predictor of post-traumatic growth, which means that compassion across the three flows and social safeness increase post-traumatic growth. This finding suggests that individuals who feel more socially safe and connected to others, and who are able to be compassionate towards themselves, to others and that receive compassion from others in the face of suffering and adversity, reveal greater post-traumatic growth in the context of the pandemic. This is congruent with the notion that having access to caring, supportive social connections has a range of benefits for mental wellbeing [19, 20]. Social support has indeed been a primary coping strategy linked to increased post-traumatic growth and resilience for people during historical large-scale disasters [27], as well as during the current pandemic [18]. As one of the dimensions of our social connection component, compassion has been associated with greater wellbeing and resilience in the face of adversity [53, 61, 115]. In particular, our study expands on current evidence revealing that self-compassion is associated with greater post-traumatic growth in the context of traumatic events [62, 63].

Interestingly, when controlling for the effect of social connection, the social disconnection component did not significantly predict post-traumatic growth, revealing that fears of compassion and loneliness are not associated with post-traumatic growth in relation to the pandemic. This is a novel finding since, although fears of compassion [64] and lack of social support [7] have been associated with PTSD during the COVID-19 crisis, no previous research has explored their relationship to post-traumatic growth. Thus, our findings suggests that, in the context of the current pandemic, social connection (i.e., compassion and social safeness) emerges as the key predictor of post-traumatic growth.

There was a significant and positive moderator effect of social connection on the impact of perceived threat of COVID-19 and post-traumatic growth. This effect was consistent across all 21 countries and was not affected by differences in questionnaire responses between countries. This is a novel and important finding that suggests that, in the context of the pandemic and across countries, one’s ability to activate compassion motivational systems across the three flows, and to experience social safeness and connectedness to others strengthens the impact of perceived threat of COVID-19 on post-traumatic growth in the face of pandemic threat. This is in line with our hypothesis that the social connection component (i.e., the compassion flows and social safeness) would magnify the effects of perceived threat of COVID-19 on recovery and growth during the pandemic. Our results build upon extensive literature on the benefits of caring supportive social connections for mental wellbeing [19–21], and for post-traumatic growth and resilience in the context of other large-scale disasters [27], or during the COVID-19 crisis [18, 116]. In particular, identification with humanity, beliefs about a good world, and openness to the future were found to be associated with post-traumatic growth during the COVID-19 pandemic [16]. This seems to be congruent with our data suggesting that when individuals are able to feel socially safe in the world and connected to others, and activate compassion motivational systems, this facilitates their resilience and growth in the face of trauma. Our findings are also in accordance with a recent study which found that self-compassion and receiving compassion from others buffer the impact of perceived threat of COVID-19 on psychological distress and social safeness [71]. In support, other studies have documented the protective role of compassion [53, 117] and social safeness [22, 23, 28] against psychological distress and as promoters of wellbeing and resilience in the face of adversity.

As expected, social disconnection was found to negatively moderate the impact of perceived threat of COVID-19 on post-traumatic growth. This finding reveals that fears, blocks and resistances to giving and receiving compassion, along with experiences of physical and emotional loneliness, significantly diminish the possibility of post-traumatic growth in the face of perceived threat of COVID-19. This novel finding expands the current evidence base and suggests that in the absence of social connection post-traumatic growth is hampered. Taken together, these results highlight that it is the social connection component, in particular compassion across the three flows and feelings of social safeness, that seems to be key to promote post-traumatic growth as an adaptive coping mechanism or as an outcome of positive psychological change in the face of a threatening event such as the current pandemic, while social disconnection may inhibit such growth.

### Post-traumatic stress

Perceived threat of COVID-19 emerged as the strongest predictor of increased post-traumatic stress. This corroborates the proposition that, exposure to the COVID-19 pandemic and to its multifaceted consequences, can be a potentially traumatic event and trigger PTSD symptomatology [10]. This finding is in accordance with mounting research demonstrating that PTSD is an outcome of the COVID-19 pandemic [6, 7], and with epidemiological studies reporting the experience of PTSD symptoms amongst the general adult population during the early stages of the pandemic [8]. Consistent with our results are also several studies establishing a link between fears of COVID-19 and indicators of poor mental health [72, 92, 118–120], in particular PTSD symptoms [121].

Interestingly, perceived threat of COVID-19 was not only a predictor of increased post-traumatic stress symptoms, but also a predictor of greater post-traumatic growth. This finding could be understood in light of previous research which describes how perceiving an event as threatening and severe can influence both the development of post-traumatic stress and post-traumatic growth [17]. This suggests that both traumatic symptoms and growth may occur because of the suffering produced by a highly stressful event, such as the COVID-19 pandemic [16].

Social disconnection predicted higher levels of post-traumatic stress, revealing that being fearful of compassion and feeling lonely and disconnected from others increased traumatic stress symptoms in the context of the current pandemic. Furthermore, in line with our hypothesis, social disconnection positively moderated the impact of perceived threat of COVID-19 on post-traumatic stress. This is a key finding which indicates that in the pandemic context, fears of receiving (from oneself and others) and giving compassion and loneliness heighten the impact of perceived threat of COVID-19 on symptoms of post-traumatic stress, across all countries.

It is well established that feeling socially disconnected and lonely, and being resistant to or afraid of compassion, are major vulnerability factors for mental health problems [74, 87, 122]. In fact, previous evidence has shown that a lack of social support is one of the best predictors of PTSD [123, 124]. This is particularly relevant under the unique circumstances of pandemic threat, where beyond the threat of the virus itself, the (almost) universal preventive containment and social distancing measures used to control the spread of the virus, have deprived people from one of the most powerful physiological and psychological regulators of threat–access to supportive social relationships [27, 74, 77]. Extensive research has confirmed that lockdown has increased experiences of social disconnection, loneliness and psychological distress [1, 2, 27, 78–81]. Studies have additionally revealed that, during the pandemic, loneliness and lack of social support are associated with greater mental health difficulties [27, 80], and that suspiciousness, which is typically linked to a lack of interpersonal trust and to low perceived social support, is related to post-traumatic stress symptoms and impairment [16]. Consistent with our data, Matos et al [72] demonstrated that fears of compassion were not only associated with greater psychological distress, but they also magnified the impact of perceived threat of COVID-19 on symptoms of depression, anxiety and stress, and that fears of receiving compassion from others amplified the negative effect of threat of COVID-19 on social safeness. Our data extends previous studies on the mediating role of fears of compassion between early emotional trauma and symptoms of depression, anxiety and paranoid ideation [88], and on loneliness as a major vulnerability factor in the context of trauma [125, 126].

Therefore, if under the pandemic threat one is afraid, resistant or unable to activate compassionate motivational systems across the three flows or use caring relationships as affect-regulators [85], then they may lack vital coping mechanisms and be unable to psychologically and physiologically regulate threatening internal (e.g., thoughts, emotions, bodily sensations) and external (e.g., someone close or oneself getting the virus, financial difficulties, work stresses) experiences. Thus, one may be more vulnerable to experience post-traumatic stress in relation to the pandemic threat, including intrusions, hyperarousal and avoidance symptoms.

Surprisingly, social connection (albeit with a smaller effect than social disconnection) predicted increased levels of post-traumatic stress, suggesting that compassion and social safeness may increase traumatic symptoms in the face of pandemic threat. However, there was no significant moderator effect of social connection on the relationship between perceived threat of COVID-19 and post-traumatic stress symptoms. A possible explanation for this finding might be the loss of social relationships, and fears for others health and wellbeing due to the unique nature of the pandemic threat and its’ associated containment and lockdown measures. Unlike other mass disasters where people used social connection and support as a way of coping with adversity and regulating threat, the current pandemic can be regarded as a form of social trauma where, although faced with a global, unpredictable and highly threatening situation, people were unable to come together, feel socially safe and supported by others, and give and receive care and compassion [27, 56]. This was especially the case at the beginning of the pandemic, when the data was collected across countries. In this period, this universal and unprecedented event was presented by authorities as a very high and unpredictable threat, with no solution in sight, other people were seen as a threat for contagion, and through the media people were faced with daily high figures of human losses, overwhelmed healthcare services and horrifying images of mass graves. Thus, at the onset of the COVID-19 crisis when people felt especially threatened, they were deprived of the possibility to socially connect and feel safe with others, to receive care, support and compassion from others and also to connect, care and be compassionate to others in the face of suffering. Hence, this pandemic context might have represented a blockage to the enactment of compassionate motivational systems, and so the more socially connected and compassionate one was, the more vulnerable one felt (in relation to oneself and others), and the greater the traumatic stress associated with the pandemic. Similarly, Vazquez et al. [16] found that beliefs of identification with humanity (which seems to be linked to a sense of common humanity related to compassion and social safeness) predicted both post-traumatic growth and post-traumatic symptoms at the beginning of the pandemic. Therefore, it seems that dimensions of social connection related to feeling socially safe and being able to give and receive compassion (from oneself and form others) may have double-edged consequences under the pandemic threat, by promoting growth and resilience, but also by increasing one’s sense of vulnerability and social loss.

### Limitations and future directions

As with any multinational study there may be differences across countries which can affect the results. In this case the differences in rates of COVID-19 and Government responses to the pandemic may affect variables such as psychological distress and the amount of social contact people receive in different countries. It is therefore a strength of this study that the results were found to be consistent across countries. Also, it is important to note that convenience samples were used and, therefore, these are not representative of the countries’ populations which may limit their generalizability. For example, more female participants consented to take part in the study (meta-analysis revealed higher self-compassion in males [127]), and there was no representation from the continent of Africa. The fact that it was an online study may also diminish its representativeness, especially among older adults, or in deprived populations without access to the internet. Thus, in the future research should attempt to recruit more men and assure an equal representativeness of different age groups, social economic strata, and greater efforts should be made to collect data across all continents. Finally, the cross-sectional nature of the study prevents the establishment of causality. In addition, this study evaluated perceived traumatic stress and growth in the beginning of the pandemic and when the stressful event (i.e., the COVID-19 pandemic) was still present. It is thus possible that, for example, traumatic stress symptoms might change over time as the pandemic as well as the preventive containment measures evolve. Also, the findings regarding post-traumatic growth might be reflecting a coping mechanism that facilitates the future development of adaptive coping strategies to adversity or of longer-term outcomes such as the enduring positive changes in personality or in philosophical views of the world, as proposed by prior longitudinal studies [128, 129]. The research project that this study integrates is currently collecting longitudinal data throughout the pandemic and future research will examine these data and map the changes in post-traumatic symptoms and post-traumatic growth as well as the prospective role of social connection and disconnection, as this global situation continues to unfold. Furthermore, after the end of this pandemic, research could also seek to examine the development of posttraumatic stress symptoms and posttraumatic growth associated with COVID-19 pandemic.

### Implications

The current study sheds light on the pivotal role social connection plays on how individuals adapt and cope with the challenging worldwide COVID-19 crisis, and hence may instruct the implementation of community-based strategies to support resilience and protect mental health in this period [81], and advise pandemic planning [89]. In fact, future pandemics with similar characteristics are more likely than ever to occur again. Therefore, the knowledge gained from this study could act as a template for future events. Given that social connection (i.e., compassion across the three flows and social safeness) seems to facilitate post-traumatic growth, and social disconnection (i.e., fears of compassion and loneliness) to increase vulnerability to develop post-traumatic stress in the context of the threat experienced during the pandemic, compassion focused interventions and dissemination of compassionate strategies of public communication might be relevant to foster individual and collective resilience and reduce mental health difficulties during and following the pandemic.

In particular, individual and community-based compassion focused interventions, such as Compassion Focused Therapy (CFT; for patients) or Compassionate Mind Training (CMT; for public) [56], might be suitable approaches to cultivate compassion across the three flows, reduce inhibitors of compassionate motivation and address fears of compassion, and promote social safeness and wellbeing in these challenging times. The benefits and efficacy of these approaches in decreasing psychological distress and promoting wellbeing in a range of populations and conditions have been widely demonstrated [86, 130, 131, for reviews]. In fact, compassion-focused interventions were found to mitigate psychological distress in the specific context of the pandemic [132, 133]. Thus, offering greater access to individual and/or group CFT and CMT, including via Telehealth, might be pertinent to promote growth and resilience, and reduce psychological distress in this context. Besides, it might be relevant to promote social reconnection amongst the general population and, in particular, vulnerable groups (e.g., elderly, health professionals), for example using community-based interventions targeting loneliness and isolation.

Additionally, public health and Government organizations could consider the implementation of strategies and communications that promote feelings of social connection and safeness and foster giving and receiving compassion, whilst reducing resistances to compassion and experiences of social disconnection and loneliness. For example, prosocial public health messaging was found to lead to greater compliance with COVID-19 lockdown measures, compared with threatening messages [134, 135]. Thus, authorities and policy makers may want to consider the way they communicate measures such as ‘social distancing’ and ‘lockdowns’ to reduce the amount of social disconnection individuals might experience as result of this messaging. Indeed, it has been proposed the use of the term ‘safe relating’, which would involve appropriate physical distancing and other precautions but where the psychological focus was on both how to create ‘safeness’ and the importance of ‘relating’ rather than distancing [56, 136]. The adoption of these strategies focused on social connection processes might not only facilitate citizens adherence to preventive and managing pandemic measures (e.g., adherence to COVID-19 vaccines), but also promote resilience and mental wellbeing during and following the pandemic.

While we are still far from understanding the full extent of the long-term effects of the COVID-19 pandemic on mental health and psychosocial wellbeing, it is possible that under this challenging context lies the possibility for individual and collective positive growth and resilience [137]. The implementation of compassion-focused strategies and interventions that cultivate social connection could support wellbeing and growth, not only for individuals, but also for families, schools, and workplaces during a pandemic.

## Conclusion

Historically social connection has been one of the main ways humans have coped with large-scale threatening events and disasters. In the context of the COVID-19 pandemic, lockdowns have deprived people from one of the most powerful physiological and psychological regulators of threat–access to supportive social relationships. This multi-national study across 21 countries revealed that social connection (i.e., compassion and social safeness) increased the likelihood of post-traumatic growth in the context of the threat people felt during the pandemic. However, social connection also increased the likelihood of experiencing post-traumatic symptoms and this may be due to a physical loss of social connection (through lockdowns) and fears for the safety of others during the pandemic. Social disconnection (i.e., fears of compassion and loneliness) increased post-traumatic stress and magnified the impact of the perceived threat of COVID-19 on traumatic symptoms. Future research should seek to map the relationship between social relating and post-traumatic growth and trauma symptoms as the pandemic situation continues to develop. Compassion focused interventions and communications could be implemented to foster a sense of social connection and cultivate compassion across the three flows, thus facilitating post-traumatic growth and resilience and protecting mental health during and in the aftermath of the global COVID-19 crisis.

## Supporting information

S1 Table Results of the CHULL test for within-model, positive aspect. (DOCX)

S2 Table Results of the CHULL test for within-model, social disconnection component. (DOCX)

S1 Fig Residuals of the Multilevel Normal homoscedastic model (PTG: m1) and their distribution. (DOCX)

S2 Fig Residuals of the Multilevel Normal heteroscedastic model (PTG: m2) and their distribution. (DOCX)

S3 Fig Residuals of the Multilevel Skew Exponential Power heteroscedastic model (PTG: m3) and their distribution. (DOCX)

S4 Fig Residuals of the Multilevel Normal homoscedastic model (IES-R: n1) and their distribution. (DOCX)

S5 Fig Residuals of the Multilevel Normal heteroscedastic model (IES-R: n2) and their distribution. (DOCX)

S6 Fig Residuals of the Multilevel Skew Exponential Power heteroscedastic model (IES-R: n3) and their distribution. (DOCX)
