# Supplementary figures and images for: The role of social connection on the experience of COVID-19 related post-traumatic growth and stress
Source: PLoS One. 2021 Dec 15;16(12):e0261384. doi: 10.1371/journal.pone.0261384 (PMC8673633; doi:10.1371/journal.pone.0261384)

**S1 Table 1**

**Table 1. Results of the CHULL test for within-model, social connection component**


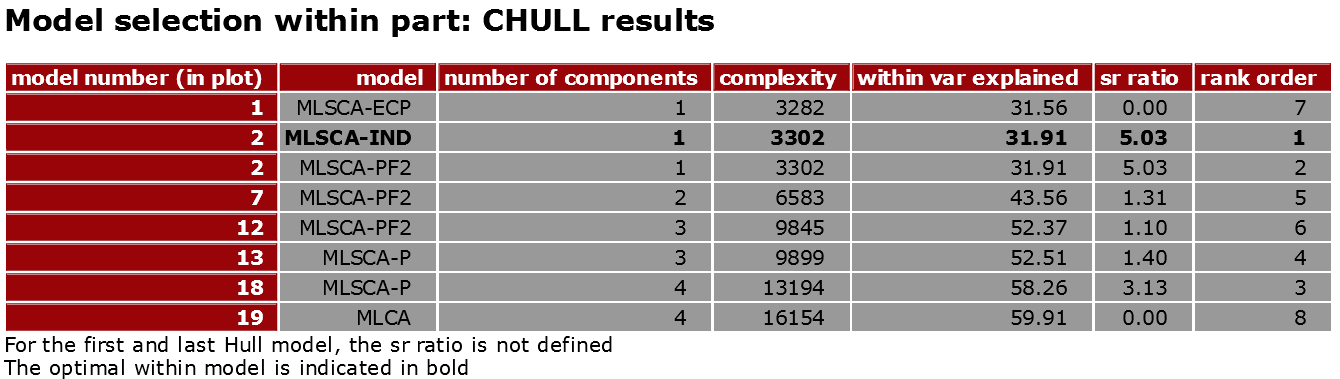

Supplement: S1 Table — (DOCX) [file pone.0261384.s001.docx]

**S2 Table 2**

**Table 2. Results of the CHULL test for within-model, social disconnection component**


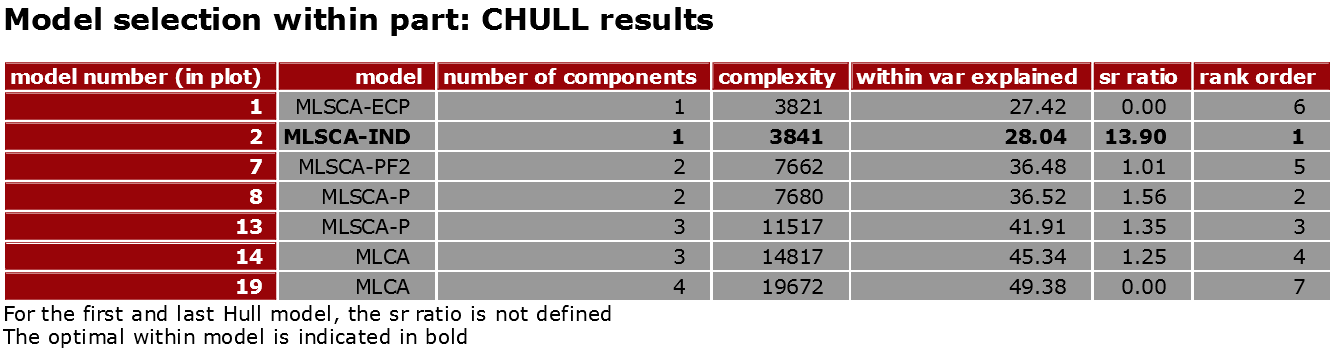

Supplement: S2 Table — (DOCX) [file pone.0261384.s002.docx]

**S3 Fig 1**

**Fig 1. Residuals of the Multilevel Normal homoscedastic model (PTG: m1) and their distribution**


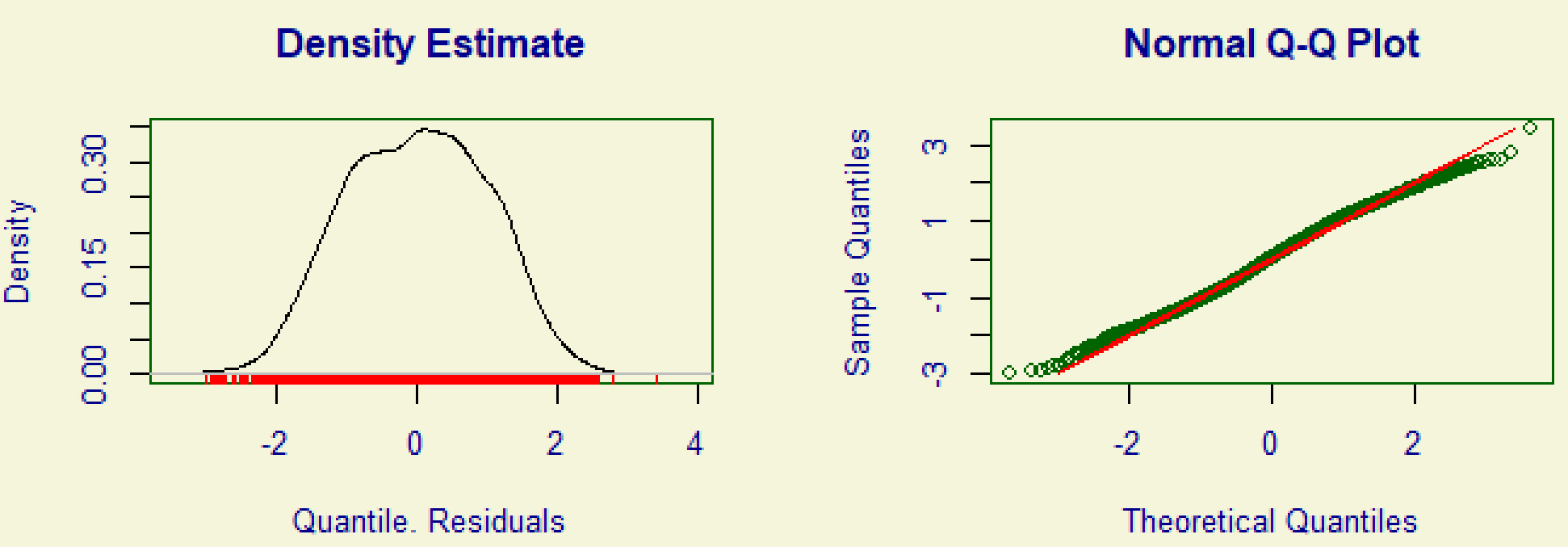


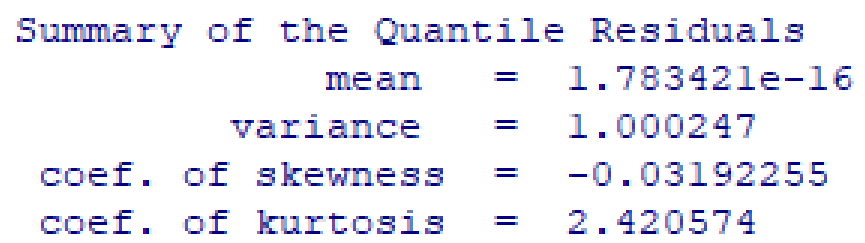

Supplement: S1 Fig — (DOCX) [file pone.0261384.s003.docx]
