# Supplementary material for: The role of social connection on the experience of COVID-19 related post-traumatic growth and stress
Source: PLoS One. 2021 Dec 15;16(12):e0261384. doi: 10.1371/journal.pone.0261384 (PMC8673633; doi:10.1371/journal.pone.0261384)

**S8 Fig 6**

**Fig 6. Residuals of the Multilevel Skew Exponential Power heteroscedastic model (IES-R: n3) and their distribution**


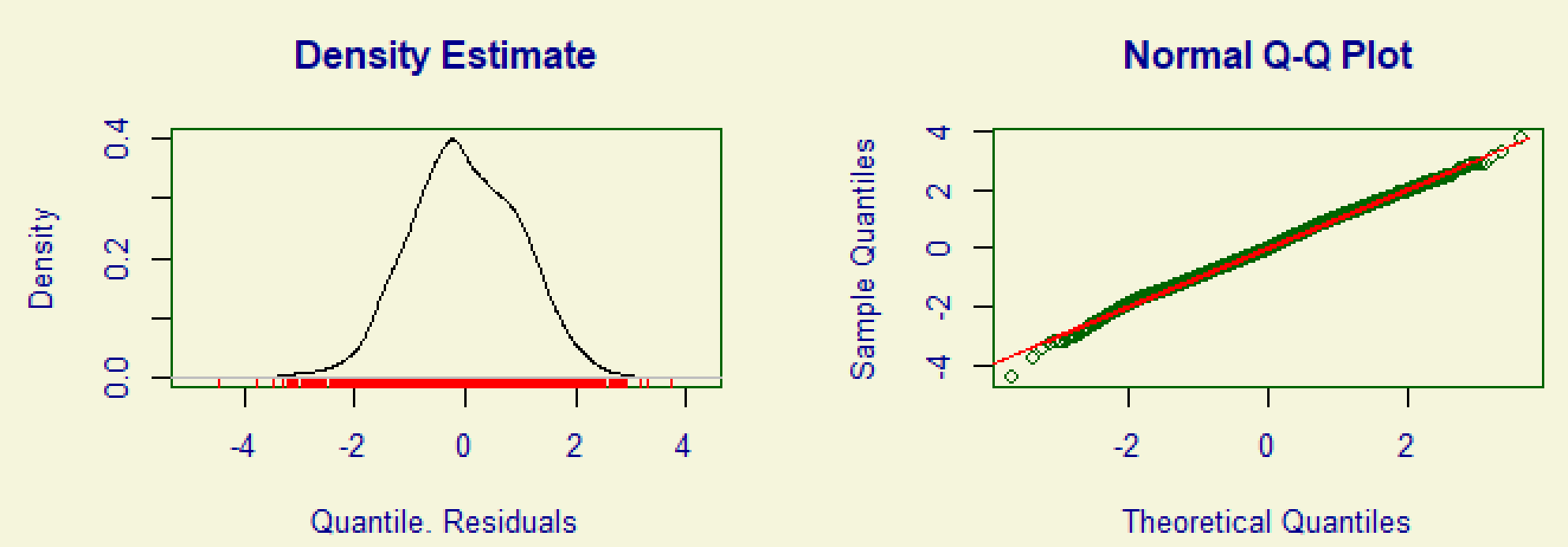


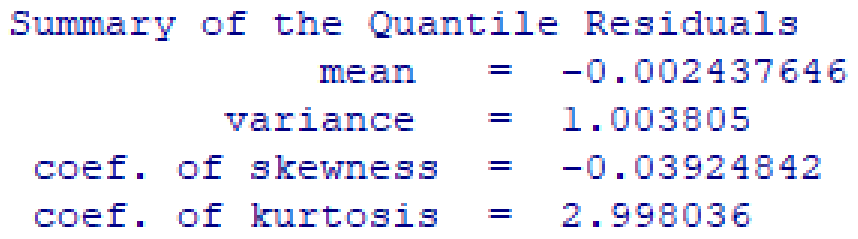

Supplement: S6 Fig — (DOCX) [file pone.0261384.s008.docx]
